# AMPA Receptors Are Involved in Store-Operated Calcium Entry and Interact with STIM Proteins in Rat Primary Cortical Neurons

**DOI:** 10.3389/fncel.2016.00251

**Published:** 2016-10-25

**Authors:** Joanna Gruszczynska-Biegala, Maria Sladowska, Jacek Kuznicki

**Affiliations:** Laboratory of Neurodegeneration, International Institute of Molecular and Cell Biology in WarsawWarsaw, Poland

**Keywords:** store-operated calcium entry (SOCE), STIM, AMPA receptors, neurons, calcium signaling

## Abstract

The process of store-operated calcium entry (SOCE) leads to refilling the endoplasmic reticulum (ER) with calcium ions (Ca^2+^) after their release into the cytoplasm. Interactions between (ER)-located Ca^2+^ sensors (stromal interaction molecule 1 [STIM1] and STIM2) and plasma membrane-located Ca^2+^ channel-forming protein (Orai1) underlie SOCE and are well described in non-excitable cells. In neurons, however, SOCE appears to be more complex because of the importance of Ca^2+^ influx via voltage-gated or ionotropic receptor-operated Ca^2+^ channels. We found that the SOCE inhibitors ML-9 and SKF96365 reduced α-amino-3-hydroxy-5-methyl-4-isoxazolepropionic acid (AMPA)-induced [Ca^2+^]_i_ amplitude by 80% and 53%, respectively. To assess the possible involvement of AMPA receptors (AMPARs) in SOCE, we used their specific inhibitors. As estimated by Fura-2 acetoxymethyl (AM) single-cell Ca^2+^ measurements in the presence of CNQX or NBQX, thapsigargin (TG)-induced Ca^2+^ influx decreased 2.2 or 3.7 times, respectively. These results suggest that under experimental conditions of SOCE when Ca^2+^ stores are depleted, Ca^2+^ can enter neurons also through AMPARs. Using specific antibodies against STIM proteins or GluA1/GluA2 AMPAR subunits, co-immunoprecipitation assays indicated that when Ca^2+^ levels are low in the neuronal ER, a physical association occurs between endogenous STIM proteins and endogenous AMPAR receptors. Altogether, our data suggest that STIM proteins in neurons can control AMPA-induced Ca^2+^ entry as a part of the mechanism of SOCE.

## Introduction

Neuronal Ca^2+^ homeostasis is precisely regulated by complex mechanisms (Berridge, 1998). Calcium ions activate various processes and are derived from the extracellular space or intracellular stores, such as the endoplasmic reticulum (ER). ER-dependent Ca^2+^ release occurs through the activation of IP_3_ receptors (IP_3_Rs) or ryanodine receptors (RyRs), leading to the depletion of Ca^2+^ stores (Berridge, 1998). Refilling these stores occurs through store-operated calcium entry (SOCE; Putney, 1986). This is the major mechanism that triggers Ca^2+^ influx in non-excitable cells (Parekh and Putney, 2005; Abdullaev et al., 2008; Sánchez-Hernández et al., 2010; Di Buduo et al., 2014). The key proteins that are involved in this process are Ca^2+^ sensors that are located in the ER (stromal interaction molecule 1 [STIM1] and STIM2) and Ca^2+^ channel-forming proteins (Orai1–3) that are present in the plasma membrane. Interactions between STIMs and Orais lead to the formation of complexes that are visible as puncta (Potier and Trebak, 2008; Shim et al., 2015) and result in Ca^2+^ entry into the cytoplasm from the extracellular space (Cahalan, 2009). STIMs can also induce Ca^2+^ influx via transient receptor potential (TRP) channels (Salido et al., 2011; Hartmann et al., 2014; Shin et al., 2016). Ca^2+^ that enters the cell is then transported to the ER through activity of the sarcoplasmic reticulum Ca^2+^-adenosine triphosphatase (SERCA) pump to refill ER stores and can be used for signaling (for review see Majewski and Kuznicki, 2015). In contrast to non-excitable cells, Ca^2+^ entry into neurons can occur via additional pathways. Two major pathways are voltage-gated Ca^2+^-channels (VGCCs) and ligand-gated Ca^2+^-channels (LGCCs), such as ionotropic glutamate receptors (Berridge, 1998; Clapham, 2007). Recent studies found that STIM–Orai-based SOCE mediates Ca^2+^ influx into cortical, hippocampal, dorsal root ganglion, and dorsal horn neurons (Klejman et al., 2009; Venkiteswaran and Hasan, 2009; Gemes et al., 2011; Koss et al., 2013; Xia et al., 2014). We found that both STIMs can form complexes with endogenous Orai1 (Gruszczynska-Biegala and Kuznicki, 2013) and are involved in Ca^2+^ homeostasis in cortical neurons. However, each STIM protein plays a distinct role in SOCE (Gruszczynska-Biegala et al., 2011; Hartmann et al., 2014). STIM1 activates SOCE when ER stores are depleted because of the activation of IP_3_Rs, whereas STIM2 has lower affinity for Ca^2+^ than STIM1, and thus is responsible for constitutive Ca^2+^ influx in resting cells (Brandman et al., 2007). We demonstrated that STIM2 in cortical neurons, in contrast to STIM1, senses and reacts to small decreases in Ca^2+^ levels in the ER that are caused by lower intracellular Ca^2+^ levels (Gruszczynska-Biegala and Kuznicki, 2013).

Recent studies found that STIM1, in addition to Orai activation, inhibits Ca^2+^ entry that is mediated by T- and L-type channels in excitable cells (Park et al., 2010; Nguyen et al., 2013). Other studies reported that STIM2 is involved in the cyclic adenosine monophosphate (cAMP)/protein kinase A (PKA)-dependent phosphorylation of the α-amino-3-hydroxy-5-methyl-4-isoxazolepropionic acid (AMPA) receptor subunit GluA1 by coupling PKA to the AMPA receptors (AMPARs) in a SOCE-independent manner (Garcia-Alvarez et al., 2015). However, no links have been established between STIM-dependent SOCE and AMPA-induced Ca^2+^ responses.

AMPARs are tetramers that are composed of homologous glutamate GluA1-GluA4 subunits in various combinations (Bigge, 1999) and are predominantly permeable for sodium and potassium ions. They also have Ca^2+^ permeability. However, when GluA2 is the dominant subunit, Ca^2+^ permeability is significantly diminished or even abolished (Hollmann and Heinemann, 1994). AMPARs are present in both neurons and glia and are involved in neuronal-glial communication in the cortex (Verkhratsky and Steinhäuser, 2000). Their subunit composition varies according to development, brain region and cell type (Song and Huganir, 2002).

In the present study, we analyzed the possible link between Ca^2+^ entry via AMPARs and SOCE in neurons. We found that SOCE was blocked by AMPAR antagonists and that SOCE inhibition decreased [Ca^2+^]_i_ rise that was induced by AMPAR agonist. These results, together with co-immunoprecipitation assays that indicated interactions between endogenous STIMs and AMPAR subunits, indicate the possible role of STIMs in AMPA-induced [Ca^2+^]_i_ changes. These observations elucidate the role of STIM proteins in the influx of Ca^2+^ via channels other than Orai and TRPs.

## Materials and Methods

### Primary Cell Cultures

Cortical neuronal cultures were prepared from embryonic day 19 (E19) Wistar rat brains. Pregnant female Wistar rats were provided by the Animal House of the Mossakowski Medical Research Centre, Polish Academy of Sciences (Warsaw, Poland). Animal care was in accordance with the European Communities Council Directive (86/609/EEC). The experimental procedures were approved by the Local Commission for the Ethics of Animal Experimentation no. 1 in Warsaw. Brains were removed from rat embryos and collected in cold Hanks solution supplemented with 15 mM HEPES buffer and penicillin/streptomycin. The cortices were isolated, rinsed three times in cold Hanks solution, and treated with trypsin for 35 min. The tissue was then rinsed in warm Hanks solution, washed three times, and dissociated by pipetting. For Ca^2+^ measurements, primary cortical neurons were plated at a density of 7 × 10^4^ cells/well on eight-well PDL-laminin-precoated chamber slides (BioCoat). For the co-immunoprecipitation assays and Western blot, neurons were seeded on poly-D-lysine-precoated BioCoat plastic Petri dishes (BD Biosciences Discovery Labware) at a density of 7 × 10^6^ cells/plate. Neurons were grown in Neurobasal medium (Invitrogen) supplemented with 2% B27 (Invitrogen), 0.5 mM glutamine (Sigma), 12.5 μM glutamate (Sigma), and a penicillin (100 U/ml)/streptomycin (100 mg/ml) mixture (Gibco). Cultures were maintained at 37°C in a humidified 5% CO_2_/95% air atmosphere. Every 3–4 days, half of the conditioned medium was removed and replaced by fresh growth medium. The experiments were performed on 15-day-old cultures (co-immunoprecipitation assay, Western blot) or 16- to 17-day-old cultures (Ca^2+^ measurements).

To make the glial cultures, a cortical cell suspension was plated on poly-D-lysine-precoated BioCoat plastic Petri dishes (BD Biosciences Discovery Labware) and grown in Dulbecco’s Modified Eagle Medium (DMEM) that contained 10% fetal bovine serum and a penicillin (100 U/ml)/streptomycin (100 mg/ml) mixture (Gibco). The cells were grown to confluence (after ~1 week) and were replated to avoid non-glial contamination. The medium was replaced once every 3–4 days. For Western blot, the cells were cultured for 15 days prior to the experiments.

### Cell Line Culture

HeLa cells were grown in DMEM that contained 10% fetal bovine serum and a penicillin (100 U/ml)/streptomycin (100 mg/ml) mixture (Gibco) at 37°C in a 5% CO_2_ atmosphere.

### Single-Cell Ca^2+^ Measurements

**Figure 1:**
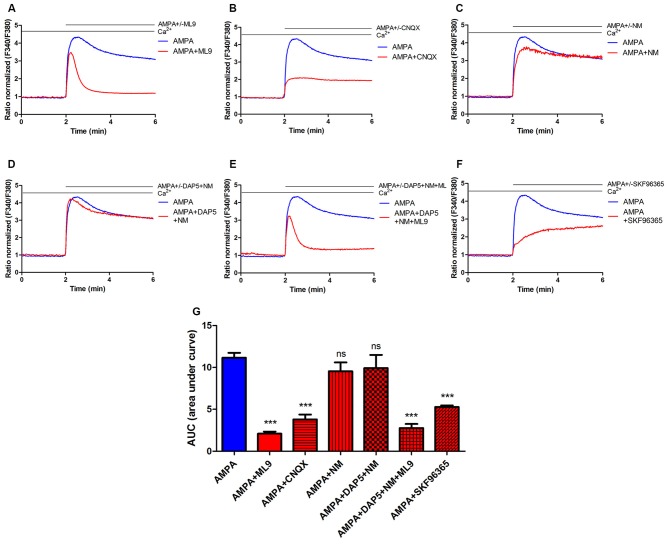
**Store-operated calcium entry (SOCE) inhibitor ML9 decreases α-amino-3-hydroxy-5-methyl-4-isoxazolepropionic acid (AMPA)-induced changes in [Ca^2+^]_i_ in rat cortical neurons. (A–F)** Average traces of AMPA-induced intracellular Ca^2+^ (F340/F380) levels obtained by ratiometric Fura-2 acetoxymethyl (AM) analysis of untreated neurons (control) and neurons treated with indicated antagonists. Measurements were started in a buffer with 2 mM CaCl_2_, then in a buffer that contained 100 μM AMPA in the absence or presence indicated antagonists 100 μM ML-9 **(A)** 30 μM CNQX **(B)** 5 μM nimodipine (NM; **C**) 10 μM DAP-5 + 5 μM NM **(D)** 10 μM DAP-5 + 5 μM NM + 100 μM ML9 **(E)** 30 μM SKF96365 **(F)**. F340/F380 values just before the addition of the AMPAR agonist were normalized to the same values (1). The data represent *n* independent experiments that were conducted on four different primary cultures, corresponding to 960 (AMPA, *n* = 17), 677 (AMPA + ML9, *n* = 13), 311 (AMPA + CNQX, *n* = 9), 309 (AMPA + NM, *n* = 10), 258 (AMPA + DAP5 + NM, *n* = 8), 289 (AMPA + DAP5 + NM + ML9, *n* = 8) and 95 (AMPA + SKF96365, *n* = 6) analyzed cells that responded to KCl. **(G)** Summary data showing AMPA-induced changes in [Ca^2+^]_i_ in treated neurons compared with untreated neurons. The data are expressed as the AUC, which was calculated from the moment immediately before the addition of Ca^2+^. ***p < 0.001; ns, not significant compared with the control (Student’s *t*-test, Mann-Whitney *U* test).

**Figure 2:**
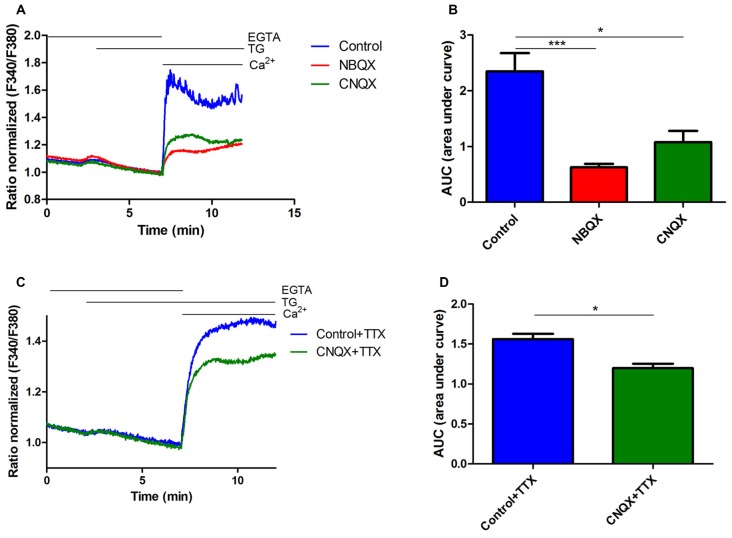
**Inhibition of thapsigargin (TG)-induced SOCE in rat cortical neurons by NBQX and CNQX. (A)** Average traces of intracellular Ca^2+^ (F340/F380) levels obtained by ratiometric Fura-2 AM analysis of neurons treated with 30 μM NBQX or 30 μM CNQX and untreated cultures (blue). Measurements were started in a medium with 0.5 mM ethylene glycol tetraacetic acid (EGTA), which was then replaced by a medium with 0.5 mM EGTA and either 2 μM TG + 30 μM NBQX or 2 μM TG + 30 μM CNQX. Finally, 2 mM CaCl_2_ was added to the medium to detect SOCE with either 30 μM NBQX or 30 μM CNQX. F340/F380 values just before the addition of Ca^2+^ were normalized to the same values (1). The data represent *n* = 28 (control), *n* = 17 (NBQX), and *n* = 13 (CNQX) independent experiments that were conducted on three different primary cultures, corresponding to 1160, 863, and 516 analyzed cells that responded to KCl, respectively. **(C)** Average traces of intracellular Ca^2+^ (F340/F380) levels obtained by ratiometric Fura-2 AM analysis of neurons treated with 30 μM CNQX + 1 μM TTX (green) and control cultures + 1 μM TTX (blue). The data represent *n* = 3 (control + TTX), and *n* = 3 (CNQX + TTX) independent experiments that were conducted on one primary culture, corresponding to 64 and 72 analyzed cells that responded to KCl, respectively. **(B,D)** Summary data showing SOCE as the AUC, which was calculated from the moment immediately before the addition of Ca^2+^. ***p < 0.001, *p < 0.05, compared with the control [Student’s *t*-test, Mann-Whitney *U* test **(B)**; *t*-test, unpaired test **(D)**].

Single-cell Ca^2+^ levels in cortical neurons were recorded using the ratiometric Ca^2+^ indicator dye Fura-2 acetoxymethyl ester (Fura-2 AM). Cells were grown on eight-well chamber slides and loaded with 2 μM Fura-2 AM for 30 min at 37°C in Hanks Balanced Salt Solution (HBSS) that contained 145 mM NaCl, 5 mM KCl, 0.75 mM Na_2_HPO_4_, 10 mM glucose, 10 mM HEPES (pH 7.4) and 1 mM MgCl_2_ supplemented with 2 mM CaCl_2_ at 37°C (high Ca^2+^ medium) and then rinsed and left undisturbed for 30 min at 37°C to allow for de-esterification. Measurements of intracellular Ca^2+^ levels were performed every 1 s at 37°C using an Olympus Scan^∧^R&Cell^∧^R imaging system that consisted of an IX81 microscope (Olympus, Tokyo, Japan), 10× 0.40 NA UPlanS Apo objective (Olympus, Tokyo, Japan), and Hamamatsu EM-CCD C9100-02 camera (Hamamatsu Photonics K.K., Hamamatsu City, Japan). Changes in intracellular Ca^2+^ concentration ([Ca^2+^]_i_) in individual neuronal cell bodies are expressed as the F340/F380 ratio after subtracting background fluorescence. This ratio represents the emission intensities at 510 nm obtained after excitation at 340 and 380 nm. The low Ca^2+^ medium (Ca^2+^-free solution) contained 0.5 mM ethylene glycol tetraacetic acid (EGTA) in the standard buffer. At the end of the experiments, 50 mM KCl in the presence of 2 mM CaCl_2_ was added to assess which cells were neurons (Figures 1, 2). Cells that responded with rapid, high [Ca^2+^]_i_ rise were identified as neurons and only these cells were analyzed in the experiments (Figures 1, 2). Approximately 60% of cells responded to KCl, which is in line with previous studies (Orlandi et al., 2011). Cells that responded with a delay or did not respond at all were assumed to be non-neuronal glial cells, most likely astrocytes. Data processing was performed using Olympus Cell^∧^R software.

### Co-Immunoprecipitation and Western Blot

For the co-immunoprecipitation of endogenous STIM1, STIM2, GluA1, and GluA2 proteins, 15-day-old primary cortical neurons that were grown on Petri dishes were washed with cold phosphate-buffered saline (PBS), scraped and centrifuged at 1000× g for 5 min at 4°C. The pellet was suspended in lysate buffer, pH 7.5, that contained 50 mM Tris-HCl, 150 mM NaCl, 0.1% sodium dodecyl sulfate (SDS), 0.5% sodium deoxycholate, 1% NP-40, and 1 mM phenylmethylsulfonyl fluoride supplemented with complete Ethylenediaminetetraacetic acid (EDTA)-free protease inhibitor cocktail (Roche) and lysed with an insulin syringe (18×). After incubation for 2 h on ice and centrifugation at 15,000× g (neuronal lysates) for 20 min at 4°C, cleared lysates were pre-incubated with 30 μl of washed protein A-Sepharose (Roche) for 3 h at 4°C. Sepharose resin and bound components were recovered by centrifugation at 15,000× g for 20 min at 4°C. Precleared lysates were subsequently incubated overnight at 4°C on a rocking platform with 30 μl of A-Sepharose that was pre-incubated earlier for 3 h with 3 μg of antibody (anti-STIM1, BD Transduction Laboratories or ProSci Inc., Poway, CA, USA; anti-STIM2, Santa Cruz Biotechnology, Santa Cruz, CA, USA; anti-GluA1, Abcam, UK; anti-GluA2, ProteinTech Group or Alomone Labs). As a negative control when indicated, lysates were omitted or were incubated with anti-Flag (Sigma) or anti-IgG antibody (Sigma). Precipitated samples were then washed three times with repeated centrifugation, eluted in 50 μl of 2× Laemmli Buffer. Protein extracts were resolved by 6% SDS-polyacrylamide gel electrophoresis (PAGE), transferred to a Protran nitrocellulose membrane (Whatman), and blocked for 2 h at room temperature in TBST (50 mM Tris-HCl [pH 7.5], 150 mM NaCl, and 0.1% Tween 20) plus 5% dry non-fat milk. Nitrocellulose sheets were then incubated in blocking solution with primary antibodies against STIM1 (1:200, ProteinTech Group), STIM2 (1:100, Alomone Labs), GluA1 (1:400, Merck Millipore) and GluA2 (1:300, ProteinTech Group) at 4°C overnight. The appropriate horseradish peroxidase-conjugated secondary antibody IgG (Sigma) was added at a dilution of 1:10,000 for 1 h. The peroxidase was detected with a Chemiluminescent Substrate Reagent Kit (Novex ECL, Invitrogen).

On Western blot containing glial cells extracts, the optical density of the bands was estimated using a GS-800 Calibrated Densitometer and Quantity One software (Bio-Rad). GAPDH was run to normalize the protein loading.

### Statistical Analysis

The statistical analysis was performed using Prism 5.02 software (GraphPad, San Diego, CA, USA). All of the data are expressed as mean ± standard error of the mean (SEM), and differences were considered significant at *p* < 0.05. Statistical significance was assessed using the nonparametric Mann-Whitney *U* test for comparisons between the mean values of unpaired groups. All of the experiments were performed at least in triplicate.

## Results

### AMPA-Induced Changes in [Ca^2+^]_i_ are Sensitive to the SOCE Inhibitors ML-9 and SKF96365

The SOCE inhibitor ML9 was used to determine whether AMPA-induced [Ca^2+^]_i_ responses are affected. Rat cortical neurons were loaded with the Fura-2 AM Ca^2+^ indicator in 2 mM CaCl_2_-containing medium, and the Ca^2+^ signal was recorded. The cells were then treated with 2 mM Ca^2+^ medium supplemented with 100 μM AMPA in the absence or presence of 100 μM ML-9. After the addition of AMPA, an increase in [Ca^2+^]_i_ changes was observed in both neuronal cultures (Figure 1A). In control neurons, a long-lasting peak in cytosolic Ca^2+^ was observed with the AMPA stimulus. However, in the presence of ML-9, the [Ca^2+^]_i_ rise was lower and decreased to basal Ca^2+^ levels after approximately 1 min (Figure 1A). The data (expressed as the area under the curve [AUC]) revealed that AMPA-induced [Ca^2+^]_i_ amplitudes decreased by 80% in the presence of 100 μM ML-9 (Figure 1G).

We next sought to determine whether AMPA-induced elevation in [Ca^2+^]_i_ in cortical neurons involves other receptors or channels. Neither the VGCC blocker nimodipine (NM; Figures 1C,G) nor *N*-methyl-D-aspartate (NMDA) receptor antagonist DAP-5 in the presence of NM (Figures 1D,G) prevented AMPA responses. However, changes in [Ca^2+^]_i_ were highly abolished (by 66%) by the AMPA/kainate receptor antagonist CNQX (Figures 1B,G). To reduce the effects of other channels on [Ca^2+^]_i_ responses, selective VGCC or NMDA receptor (NMDAR) blockers were applied in the presence of ML-9. Figure 1E shows that AMPA-induced [Ca^2+^]_i_ responses were reduced by ML-9 in the presence of NM and DAP-5 by 75%, thus confirming its inhibitory effects. To further examine the impact of SOCE on AMPA-induced [Ca^2+^]_i_ response we used another SOCE inhibitor—SKF96365. In the presence of SKF96365 the AMPA-induced changes in [Ca^2+^]_i_ were decreased by 53% (Figures 1F,G). All these results suggest that intracellular [Ca^2+^]_i_ elevations after AMPA stimulation in neuronal cultures occur primarily through AMPARs and store-operated channels and not through NMDARs or voltage-gated ion channels.

### SOCE in Neurons is Decreased by AMPA Receptor Antagonists

Since the SOCE inhibitors blocked AMPA-induced [Ca^2+^]_i_ rise, we next investigated whether AMPAR activity participates in SOCE. We used the potent AMPAR antagonists CNQX and NBQX. Ca^2+^ recordings were performed in rat cortical neurons. Cells were loaded with the Fura-2 Ca^2+^ indicator in 2 mM CaCl_2_-containing medium and then incubated with 0.5 mM EGTA medium to initiate the measurements. The neuronal cultures were then treated with thapsigargin (TG) and either NBQX or CNQX. After 5 min, a medium with 2 mM CaCl_2_ was added to induce SOCE in the presence of 30 μM NBQX or 30 μM CNQX. To distinguish neurons from other cells that were present in the culture, KCl was added at the end of the experiments, and the Ca^2+^ signal was recorded (data not shown). Only cells that responded with fast and transient increases in Ca^2+^ were considered neurons (Figure 2). To eliminate the possible effect of synapses activation on SOCE, we used 1 μM tetrodotoxin (TTX), which prevents firing. The addition of Ca^2+^ to the medium increased intracellular Ca^2+^ levels under all of the tested conditions (Figure 2A). These effects were substantially blunted by exposing the neurons to the AMPAR antagonists. Treatment with NBQX and CNQX suppressed TG-induced SOCE in neurons by 73% and 55%, respectively, compared with control cells (Figure 2A), reflected by a decrease in the AUC (Figure 2B). SOCE responses were also reduced by CNQX in the presence of TTX by 23% (Figures 2C,D). These results suggest that AMPARs are involved in SOCE in rat cortical neurons and may be activated after TG-induced Ca^2+^ store depletion.

### Differential Effects of NBQX on SOCE in Glia and Neurons

**Figure 3:**
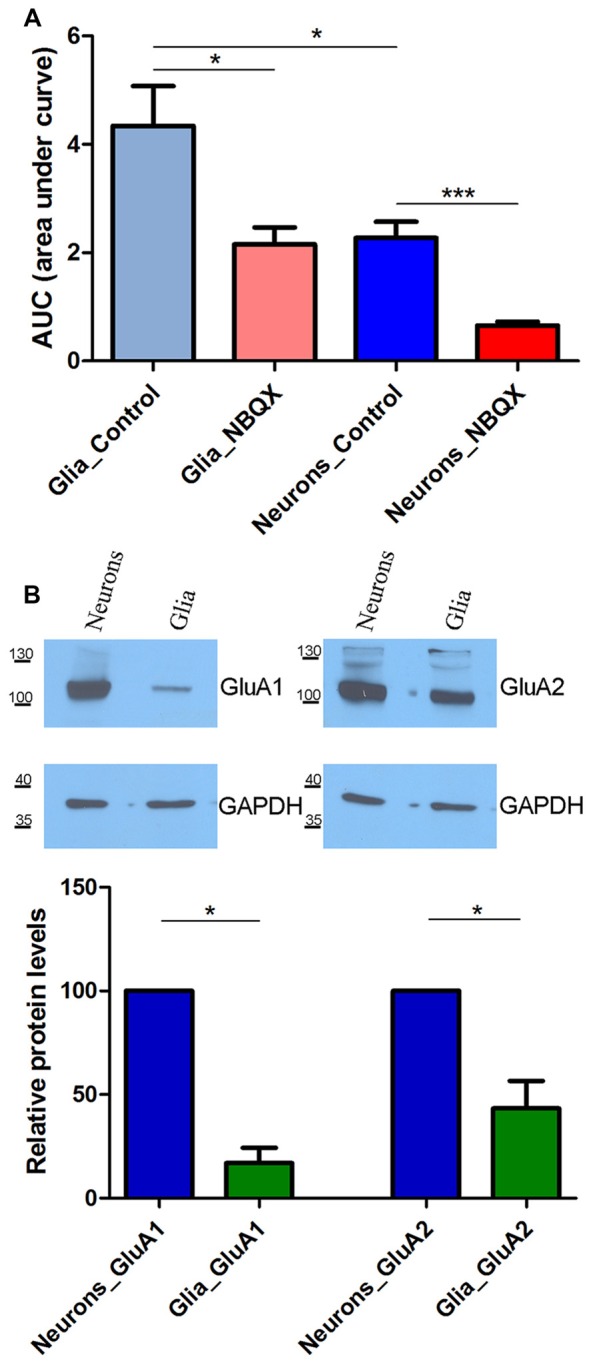
**SOCE analysis in glia and neurons in the presence of 30 μM NBQX. (A)** Experiments were performed as described in Figure 2 SOCE is shown as the AUC, which was calculated from the moment immediately before the addition of Ca^2+^. The number of recorded cells was 470 glial cells, 503 glial cells in the presence of NBQX, 869 neurons, and 863 neurons in the presence of NBQX. ***p < 0.001, *p < 0.05 (Student’s *t*-test, Mann-Whitney *U* test). **(B)** Expression of AMPA receptors (AMPARs) GluA1 and GluA2 subunits in whole-cell cortical lysates from neurons and glia. GAPDH was used as a loading control. The molecular masses of the markers that were run on the same gel are shown on the left. Bars indicate the quantification of Western blots showing the GluA1 and GluA2 levels normalized to the level of GAPDH. Values are expressed as a percentage of protein levels in neurons. The blots were performed four times from four independent cultures. *p < 0.05 (Student’s *t*-test, Mann-Whitney *U* test).

In the above experiments, cells in culture that did not respond to KCl were considered glial cells. The data that were collected for these cells were also analyzed (Figure 3A). Consistent with our previously published results (Steinbeck et al., 2011), TG-induced SOCE was higher in glial cells than in neurons, with an approximately two-fold difference in SOCE (indicated by the AUC). Glial SOCE was decreased by 46% by 30 μM NBQX, whereas neuronal SOCE was decreased by 73% in these cultures (Figure 3A).

We next investigated whether this difference can be explained by the expression of different AMPAR subunits in neurons and glia. To examine the protein expression of the AMPAR GluA1 and GluA2 subunits, Western blot was performed using lysates from cultures of neuronal and glial cells that were grown separately. Both GluA1 and GluA2 were detected in neurons. In glial cells, they markedly decreased or were barely detectable (Figure 3B). The densitometric analysis of the band intensities in four experiments indicated that there was 5.9 and 2.3 times more GluA1 and GluA2 in neurons than in glia, respectively (*p < 0.05; bars on Figure 3B). These data indicate that neuronal SOCE is more sensitive to NBQX than glial SOCE, likely because of the higher expression of AMPAR subunits in neurons.

### No Effect of AMPAR Antagonists on SOCE in HeLa Cells

**Figure 4:**
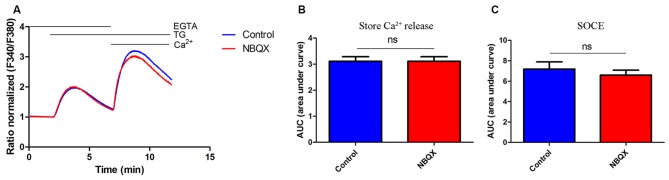
**NBQX did not block TG-induced SOCE in HeLa cells. (A)** Average traces of intracellular Ca^2+^ (F340/F380) levels obtained by ratiometric Fura-2 AM analysis of cells treated with 30 μM NBQX (red line) and untreated cells (blue line). Measurements were conducted as described in Figure 2. F340/F380 values just before the addition of TG were normalized to the same values (1). The data represents 16 independent measurements conducted in three different experiments, corresponding to 1300 for control cells and 1000 for NBQX cells. **(B,C)** Summary data presented as the AUC, showing TG-induced Ca^2+^ release from the endoplasmic reticulum (ER), which was calculated from the moment of the addition of TG until extracellular Ca^2+^ was added **(B)** or SOCE, which was calculated from the moment immediately before the addition of Ca^2+^ **(C)**. ns, not significant (Student’s *t*-test, Mann-Whitney *U* test).

To exclude the possibility that NBQX inhibits SOCE by interacting with the STIM/Orai complex, we tested its effect in HeLa cells, which lack AMPARs. The treatment of HeLa cells with 30 μM NBQX did not affect the average peak of TG-induced Ca^2+^ release (Figure 4A, first peak) and only slightly decreased SOCE (Figure 4A, second peaks). The observed difference was not statistically significant (Figures 4B,C). These results indicate that NBQX does not inhibit SOCE in neurons directly but rather inhibits SOCE by decreasing AMPAR activity.

### STIM1 and STIM2 Interact with AMPA Subunits in Rat Cortical Neurons

**Figure 5:**
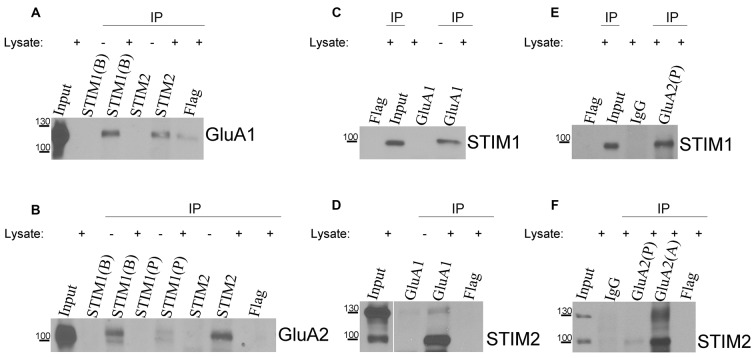
**Co-immunoprecipitation of endogenous stromal interaction molecule 1 (STIM1) and STIM2 with GluA1 and GluA2 in lysates of neuronal cultures.** Neurons were treated with TG for 10 min and lysed. Proteins were immunoprecipitated with **(A,B)** anti-STIM1 (BD Transduction Laboratories [B], ProSci [P]), **(A,B**) anti-STIM2, **(C,D)** anti-GluA1, or **(E,F)** anti-GluA2 (Proteintech [P], Alomone [A]) antibodies. Lysates (Inputs) and eluted fractions (immunoprecipitates, IP) were separated on 6% sodium dodecyl sulfate (SDS)-PAGE gels, and proteins were identified by Western blot using anti-GluA1, GluA2, STIM1, or STIM2 antibodies as indicated. When indicated, the lysates were omitted in the probes. Irrelevant mouse or rabbit anti-Flag and/or rabbit anti-IgG antibodies were used as negative controls. The molecular masses of the markers that were run on the same gel are shown on the left. Immunoprecipitation was performed three times from three different cultures with the same positive outcome.

The data above suggest a relationship between AMPARs and SOCE. To confirm the observed participation of AMPARs in [Ca^2+^]_i_ elevation under SOCE conditions, we evaluated whether endogenous STIM proteins interact with endogenous AMPAR subunits. We performed co-immunoprecipitation experiments using extracts of cultures of rat cortical neurons that were treated with TG to induce SOCE. Specific STIM1, STIM2, GluA1, and GluA2 antibodies were used, and the immunoprecipitates were analyzed by Western blot. Anti-Flag antibodies and IgG were used to identify possible nonspecific interactions. When the neuronal lysates were immunoprecipitated with the anti-STIM1 antibody, the bands of GluA1 (Figure 5A) and GluA2 (Figure 5B) were detected in the immunoprecipitates. The presence of both GluA1 and GluA2 was also detected in the immunoprecipitates of STIM2 (Figures 5A,B). Both subunits were missing in the negative control samples without the lysate. A very weak band of GluA1 and no band of GluA2 were observed in the immunoprecipitates with anti-Flag antibody. These data indicated that STIM1 and STIM2 may physically associate with AMPAR subunits in cultures of rat cortical neurons (Figures 5A,B).

In reverse experiments, in which neuronal lysates were immunoprecipitated with anti-GluA1 antibody (Figures 5C,D) or anti-GluA2 antibody (Figures 5E,F), the presence of STIM1 (Figures 5C,E) or STIM2 (Figures 5D,F) was detected. Samples with omitted lysates that were probed for STIM1 or STIM2 did not show any GluA1. No interactions were found between control IgG and STIM proteins in anti-GluA immunoprecipitates (Figures 5E,F). No STIM bands were detected in the Flag immunoprecipitates (Figures 5C–F). Our co-immunoprecipitation experiments indicate that STIM1 and STIM2 qualitatively associate with GluA1 and GluA2. This supports the calcium imaging data.

## Discussion

The major Ca^2+^ influx pathway in neurons is through VGCCs and ionotropic glutamate receptors (Verkhratsky and Kettenmann, 1996). However, previous studies also demonstrated the presence of SOCE in cultured neurons (Emptage et al., 2001; Kachoei et al., 2006; Berna-Erro et al., 2009; Klejman et al., 2009; Gruszczynska-Biegala et al., 2011). Additionally, the SOCE component STIM1 does not only activate Orai1, but it also modulates the activity of Ca_V_1.2 (Park et al., 2010; Harraz and Altier, 2014) and Ca_V_3.1 (Nguyen et al., 2013) VGCCs, the plasma membrane Ca^2+^ adenosine triphosphatase pump (Krapivinsky et al., 2011), and metabotropic glutamate one receptor-dependent TRPC3 (Hartmann et al., 2014). The results demonstrate that STIM proteins may impact various Ca^2+^-related pathways in neurons (Harraz and Altier, 2014; Kraft, 2015; Majewski and Kuznicki, 2015; Moccia et al., 2015).

The major finding in the present study was the functional relationship between SOCE, STIM proteins and ionotropic AMPARs. We showed that interactions between endogenous STIM1/STIM2 and AMPAR GluA subunits occur in cortical neurons and that SOCE-dependent mechanisms affect AMPA-mediated [Ca^2+^]_i_ rise. TG-induced SOCE was decreased 2.2–3.7 times in the presence of the potent competitive AMPAR antagonists CNQX and NBQX, suggesting that Ca^2+^ store depletion leads to Ca^2+^ influx by AMPARs. There exists a possibility that addition of calcium activates synapses, causing a large increase in network activity, and consequently an influx of calcium through NMDA and AMPARs. Indeed, Baba et al. (2003) suggested that in hippocampal pyramidal neurons SOCE can contribute to synaptic activity. We show that blocking the activity by TTX does not remove the inhibition of SOCE by CNQX, which confirms that SOCE is still mediated by AMPAR blockade (Figures 2C,D). Moreover, to minimalize an influx through the NMDAR, the Ca^2+^ imaging experiments were performed in the presence of Mg^2+^ ions, when the initial glutamate-induced [Ca^2+^]_i_ peak is severely blunted. As the membrane depolarizes, the Mg^2+^ blockage is removed (Nowak et al., 1984). However, Kachoei et al. (2006) demonstrated that the source of Ca^2+^ during SOCE appears to be solely from the SOCE pathway, given that the depolarization is too small to bring the membrane potential near the threshold for activation of the voltage-gated Ca^2+^ channels. The decrease in SOCE in neurons was attributable to the inhibition of Ca^2+^ influx through AMPARs, which was supported by observations in HeLa cells, which do not possess AMPARs, in which SOCE and Orai channels were unaffected by NBQX. We propose that the induction of SOCE by TG leads to the activation of Orai, but also to activation of AMPARs either directly through the recruitment of AMPARs by STIM proteins or indirectly through unknown mechanisms.

STIM2 was recently proposed to shape basal transmission in excitatory neurons and is involved in the formation of dendritic spines and essential for the cAMP/PKA-dependent phosphorylation of AMPARs (Garcia-Alvarez et al., 2015). These authors speculated that STIM2 might regulate various forms of synaptic plasticity by managing GluA1-PKA coupling at excitatory synapses. They also performed co-immunoprecipitation assays and reported an interaction between the AMPAR GluA1 subunit and STIM2 in rat brains and in neurons that overexpressed yellow fluorescent protein (YFP)-STIM2. Our co-immunoprecipitation results confirmed the physical interaction between GluA1 and STIM2 and further demonstrated that endogenous STIM1 interacts with GluA1, and both endogenous STIM proteins interact with GluA2. These physical interactions were observed under conditions of SOCE, suggesting that STIM-AMPAR interactions result from Ca^2+^ store depletion. Ca^2+^ imaging data show that AMPARs are functional which indicates that they are located on the plasma membrane. Previously, we demonstrated that cultures of primary cortical neurons STIM proteins were present only in the membrane fraction (but not in cytosolic fraction) and the TG-treatment increased the level of both proteins in the membrane fractions (Gruszczynska-Biegala et al., 2011). Since the co-immunoprecipitation experiments were performed after TG treatment, it seems that the STIM-AMPAR association takes place in the membrane fractions, most probably at the ER-plasma membrane junctions. STIM2 was previously shown to promote the PKA-dependent surface delivery of GluA1 (Garcia-Alvarez et al., 2015). STIM1 prevents the surface expression of Ca_V_1.2 (Park et al., 2010) and Ca_V_3.1 (Nguyen et al., 2013), but it is unknown whether STIM1 also augments the surface expression of AMPARs.

In the cerebral cortex of mammals majority of pyramidal cells (glutamatergic) contains GluA2 subunit of AMPAR, whereas non-pyramidal cells (mainly GABAergic inhibitory interneurons) preferentially express GluA1-specific mRNA (Martin et al., 1993; Jonas et al., 1994). For example, neocortical layer V pyramidal neurons contained approximately 9/75/13/3% of GluA-1/2/3/4 subunits, while neocortical layer IV non-pyramidal neurons contained 57/24/l3/6 GluA-1/2/3/4 subunits, respectively (Jonas et al., 1994). Our previous studies reported the presence of strong immunostaining for STIM1 in pyramidal neurons of layer V of the cerebral cortex. In the other cortical layers, immunostained cell profiles were present, although the intensity of the signals was weaker than that observed in layer V. Granular layer IV with majority of interneurons had lowest signal (Klejman et al., 2009; Skibinska-Kijek et al., 2009). Expression of STIM2 in the neuronal cell types of cortex has not been investigated so far. Given that in the neocortex approximately 20–30% of neurons are interneurons (Markram et al., 2004), we may speculate that the association between GluA1 or GluA2 with STIM1 or STIM2 occurs mainly in pyramidal cells. However, due to the high expression of GluA1 in non-pyramidal cells this interaction may also take place in these cells.

ML-9 is a myosin light chain kinase (MLCK) inhibitor that is commonly used to inhibit SOCE (Watanabe et al., 1996; Smyth et al., 2008; Potier et al., 2009; Gruszczynska-Biegala et al., 2011; Steinbeck et al., 2011; Li et al., 2013; Lin et al., 2016; Wang et al., 2016). We found that [Ca^2+^]_i_ amplitudes activated by AMPA decreased in the presence of ML-9. Moreover, the peak in cytosolic Ca^2+^ was transient in the presence of ML-9, in contrast to the long-lasting peak that was observed with AMPA stimulation alone, suggesting that SOC may be responsible for the prolonged AMPA-induced [Ca^2+^]_i_ responses in rat cortical neurons. The response to AMPA stimulation was unaffected by the VGCC antagonist NM alone or in combination with the NMDAR antagonist DAP-5 but was abolished when NM and DAP-5 were added together with ML-9. Thus, the SOCE antagonist ML-9 inhibited Ca^2+^ influx through AMPARs, but the mechanism of this activity is unclear. Our previous study found that ML-9 decreased SOCE by ~65% in rat cortical neurons (Gruszczynska-Biegala et al., 2011). Although the site of action of ML-9 on STIM1 remains unknown, it seems to have an inhibitory effect on SOCE by either preventing or reversing STIM puncta formation (Smyth et al., 2008). Since ML-9 is an MLCK antagonist, one possibility is that AMPAR inhibition is achieved through this mechanism. Resolving this issue is especially important because MLCK inhibitors depress NMDAR-mediated currents and NMDA miniature excitatory postsynaptic currents in hippocampal neurons (Lei et al., 2001). These observations imply that an intact cytoskeleton is required for the basal regulation of NMDARs by MLCK. However, they also indicate that MLCK does not affect AMPAR-mediated currents, suggesting that this kinase specifically modulates NMDAR function (Lei et al., 2001).

The inhibitory effect of ML-9 on SOCE is not completely understood, but it seems to be unrelated to the inhibition of MLCK. Earlier work revealed that knocking down MLCK using siRNA or wortmannin (another MLCK inhibitor) does not affect SOCE or STIM1 redistribution (Smyth et al., 2008). In HEK293 cells that overexpressed YFP-STIM1, ML-9 significantly reduced the number of STIM1-Orai1 puncta structures (Smyth et al., 2008). In primary neuronal cultures, ML-9 also decreased the number of endogenous STIM1-Orai1 puncta (Gruszczynska-Biegala and Kuznicki, 2013). Assuming that ML-9 is a modulator of STIM1 translocation, we postulated that STIM1 interacts with AMPARs. Our co-immunoprecipitation results in the present study supported this hypothesis. Since STIM2 also associates with both AMPAR subunits it is likely that this Ca^2+^ sensor is also affected by ML-9. Indeed, we previously demonstrated that ML-9 inhibits STIM2 enrichment in the membrane fraction after store depletion (Gruszczynska-Biegala et al., 2011). Some studies have shown that ML-9 also antagonizes cAMP-dependent PKA and protein kinase C at the concentration used in this study (Saitoh et al., 1987; Bain et al., 2003). To be sure that AMPA-induced [Ca^2+^]_i_ response was decreased by blockade of SOCE we applied another commonly used SOCE blocker—SKF96365 and found that it also significantly suppressed the AMPA-induced changes in [Ca^2+^]_i_. These results confirm that some of SOCE-responsive proteins are involved in AMPA-induced [Ca^2+^]_i_ rise. The discrepancy between inhibitory effect of ML-9 and SKF96365 on AMPA-induced [Ca^2+^]_i_ amplitude (Figures 1A,F) is probably due to the different selectivity of these inhibitors for SOCE. It was shown that SKF96365 is able to inhibit TRP channels as well (reviewed in Prakriya and Lewis, 2015), which could explain different kinetics of the [Ca^2+^]_i_ responses. Moreover, the mechanism by which these compounds exert their inhibitory effects could be different. Since ML-9 and SKF96365 seem to be rather STIM-mediated Ca^2+^ influx inhibitors (reviewed in Majewski and Kuznicki, 2015), it suggests their major role in the rise in [Ca^2+^]_i_ induced by AMPA. Other potential mechanism, including the plasma membrane protein Orai may also be involved. Orai may enhance STIM recruitment to the proximity of AMPARs by effectively trapping STIM in puncta as was shown in the case of Ca_V_1.2 channels (Wang et al., 2010). We cannot exclude the existence of AMPAR-STIM-Orai complex. However, it was shown recently that Orai does not interact with GluA1 in YFP-STIM2-expressing hippocampal neurons and in rat brains. This implies that the two machineries (STIM2/GluA1 and STIM/SOCE) are not necessarily coupled (Garcia-Alvarez et al., 2015). Further work will be required to establish the exact mechanism of AMPAR-STIM-Orai interaction. Another question arises, whether in glutamate-stimulated neurons, STIM/Orai1 and STIM/AMPARs work independently or interact during Ca^2+^ entry. SOCE might be a key mediator of glutamate-induced HT-22 cell injury. Recently, it was shown that in these cells the levels of STIM1 or Orai1 proteins did not change after glutamate treatment, whereas glutamate provoked a redistribution of STIM1 into puncta within the cell, and might cause STIM1-mediated calcium influx (Rao et al., 2016). Moreover, increased Homer1a protein levels significantly inhibited SOCE and decreased the association of STIM1-Orai1 triggered by glutamate. However, the detailed mechanisms of interaction between glutamate receptors and STIM proteins will have to be further studied.

It is well known that a sustained Ca^2+^ entry is linked to InsP_3_-dependent Ca^2+^ store depletion induced by activation of metabotropic glutamate receptors (Berridge, 1998). In this way glutamate activates SOCE by recruiting STIM1 and Orai1. Since we show that AMPA responses are not mediated by VGCC or by NMDARs, it would be interesting to investigate if responses to glutamate are not mediated by SOCE either. Noteworthy, in culture of rat cortical astrocytes at higher concentration of agonist additional Ca^2+^ influx pathway activated by glutamate was found. This influx pathway, attributable to AMPA/kainite receptors, is sensitive to NBQX but insensitive to SOCE blocker La^3+^ (Pizzo et al., 2001).

In the present study, we found that SOCE in neurons was approximately two-times lower than in glial cells. This seems to reflect the larger size of Ca^2+^ stores in glia, in which TG-induced SOCE should lead to greater Ca^2+^ influx than in neurons. Similar observations were described previously (Arakawa et al., 2000; Steinbeck et al., 2011). Another possibility is that the dissimilarity in TG-induced Ca^2+^ entry may be attributable to differences in SOCE channels or their mechanism of activation between glial and neuronal cells. We found that NBQX decreased glial SOCE by 46%, whereas it decreased neuronal SOCE by 73%. This likely reflects the amount and type of AMPARs in both types of cells (Figure 3B; Verkhratsky and Steinhäuser, 2000). Although glial cells possess functional AMPARs (Pizzo et al., 2001; Lalo et al., 2006), the glial population does not contribute significantly to the total amount of AMPARs when analyzed by Western blot and real-time polymerase chain reaction (Orlandi et al., 2011). The expression of all four AMPA subunits was detected in rat cortical glia, but the GluA2 subunit was most abundant (Holzwarth et al., 1994). In cortical neurons, GluA1 and GluA2 are the most highly expressed subunits at each stage of development (Orlandi et al., 2011). However, astrocytes have lower expression of Ca^2+^-permeable AMPARs with Q/R-unedited GluR2 subunits than neurons (Whitney et al., 2008). In cortical astrocytes, AMPARs are stimulated by glutamate and AMPA and can be inhibited by NBQX (Pizzo et al., 2001; Lalo et al., 2011). Glutamate-induced Ca^2+^ influx in rat cortical astrocytes was also shown to be sensitive to LaCl_3_, which inhibits SOCE (Pizzo et al., 2001). This work demonstrates that AMPARs appear to be involved in SOCE in neurons, suggesting that the inhibition of these receptors in primary cultures or in the brain would also affect glial SOCE (Figure 3A).

Recently, SOCE in neurons has become the subject of intense research with regard to disturbances in Ca^2+^ homeostasis that are observed in several neurological disorders, including Alzheimer’s disease, Parkinson’s disease, Huntington’s disease, chronic epilepsy, amyotrophic lateral sclerosis, painful nerve injury and cerebral ischemia (Leissring et al., 2000; Berna-Erro et al., 2009; Steinbeck et al., 2011; Wu et al., 2011; Czeredys et al., 2013; Kawamata et al., 2014). Because of the high expression of STIM2 in the brain (Berna-Erro et al., 2009; Skibinska-Kijek et al., 2009), lower sensitivity of STIM2 to ER Ca^2+^ store depletion, and slower oligomerization rate of STIM2 compared with STIM1 (Stathopulos et al., 2009; Hoth and Niemeyer, 2013), STIM2 appears to be the major player in the nervous system. Indeed, disturbances in the pathways that involve STIM2 lead to mushroom spine loss (Sun et al., 2014; Garcia-Alvarez et al., 2015; Popugaeva et al., 2015; Zhang et al., 2015), neuronal injury (Gemes et al., 2011; Rao et al., 2015), and deficits in spatial learning (Berna-Erro et al., 2009). Disturbances in STIM2-associated pathways have also been observed in lymphocytes in familial Alzheimer’s disease (Bojarski et al., 2009), brain tissue in ageing mice and humans who suffer from sporadic Alzheimer’s disease (Sun et al., 2014; Popugaeva et al., 2015). However, it was recently postulated that synaptopodin regulates activity-dependent Ca^2+^ signals by recruiting STIM1 and Orai1 to the postsynaptic density (Korkotian et al., 2014; Segal and Korkotian, 2014) or recruits STIM1 to the postsynaptic density to activate TRPC3 (Hartmann et al., 2014). Moreover, STIM1 inhibits the activity of Ca_V_1.2 (Park et al., 2010) and Ca_V_3.1 (Nguyen et al., 2013) VGCCs. Similar to disturbances in SOCE, AMPAR upregulation leads to excessive Ca^2+^ influx, which may be involved in the neuronal dysfunction that is observed in schizophrenia, Alzheimer’s disease, Parkinson’s disease, amyotrophic lateral sclerosis, and global ischemia (Akbarian et al., 1995; Pellegrini-Giampietro et al., 1997; O’Neill et al., 2004; Kwak and Kawahara, 2005; Marenco and Weinberger, 2006; Van Den Bosch et al., 2006). Our findings demonstrate that AMPA-mediated [Ca^2+^]_i_ responses in neurons are a part of the SOCE process, suggesting a possible relationship between SOCE, STIM proteins, and AMPARs in these diseases.

In conclusion, the present data demonstrate that SOCE is functional in neurons as an AMPA-dependent Ca^2+^ signal. Together with the studies by Park et al. (2010) and Garcia-Alvarez et al. (2015), our results describe a new role for STIM proteins and SOCE in neuronal signaling by interplaying with the various neuronal Ca^2+^ channels present in the plasma membrane.

## Author Contributions

JG-B conceived, designed and performed experiments, analyzed the data, and wrote the manuscript. MS performed the experiments, analyzed the data, and wrote the manuscript. JK designed the experiments, analyzed the data and wrote the manuscript.

## Conflict of Interest Statement

The authors declare that the research was conducted in the absence of any commercial or financial relationships that could be construed as a potential conflict of interest.
